# Model‐driven engineering of safety and security software systems: A systematic mapping study and future research directions

**DOI:** 10.1002/smr.2457

**Published:** 2022-05-26

**Authors:** Atif Mashkoor, Alexander Egyed, Robert Wille, Sebastian Stock

**Affiliations:** ^1^ Johannes Kepler University Altenbergerstraße 69/, Linz 4040 Austria; ^2^ Technical University of Munich Arcisstrasse 21 80333 Munich Germany; ^3^ Software Competence Center Hagenberg GmbH Softwarepark 32a, 4232 Hagenberg Austria

**Keywords:** model‐driven engineering, safety and security, systematic mapping

## Abstract

This article presents a systematic mapping study on the model‐driven engineering of safety and security concerns in software systems. Combined modeling and development of both safety and security concerns is an emerging field of research as both concerns affect one another in unique ways. Our mapping study provides an overview of the current state of the art in this field. This study carefully selected 143 publications out of 27,259 relevant papers through a rigorous and systematic process. This study then proposes and answers questions such as frequently used methods and tools and development stages where these concerns are typically investigated in application domains. Additionally, we identify the community's preference for publication venues and trends. The discussion on obtained results also features the gained insights and future research directions.

## Graphical abstract

This article presents a systematic mapping study on the model‐driven engineering of safety and security concerns in software systems. This study answers research questions such as frequently used methods and tools, development stages, and application domains. An overview of the overlapping between evaluation domains, development stages, and employed methods and tools within the safety and security software systems

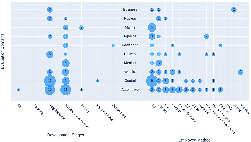

## 1 INTRODUCTION

The notion of (functional) safety is defined as freedom from risk of damage to the user, property, or environment and correct operation of a system in response to its inputs.^1^ Security, on the other hand, is the prevention of illegal access causing change and destruction of equipment, information, and services.^2^ To contrast safety and security, we say that safety aims to avoid hazards to the system's environment while security seeks to protect a system from its environment. Traditionally, depending upon the domain and the background, software engineers primarily focus on designing systems that are either safe or secure.^3^ Typically, we find many methods that investigate these concerns separately. While some exchange of ideas existed across these concerns, modern systems require their thorough, combined treatment because security concerns may affect safety and vice versa. This is particularly true for Cyber‐Physical Systems or Internet of Things (whose components communicate through networks and transcend hardware/software), where there is a good chance for safety concerns to be exposed and thus vulnerable to other types of failures caused by security concerns (or vice versa).^4^ Therefore, connected (and consequently exposed) safety systems must be equipped with security mechanisms for protection against such attacks. Only recently, there has been a surge in research focusing on the integrated modeling and development of safety and security systems.^5^

Designing software systems dealing with safety and security requires overwhelming information regarding requirements and how those are connected. The model‐driven engineering (MDE) paradigm could assist in the designing of such systems. MDE^6^ is a development paradigm that focuses on creating models which could be systematically transformed into (correct) pieces of software. The advantage is that developers can exclusively concentrate on modeling the problem rather than worrying about unnecessary and distracting implementation details. Furthermore, different MDE approaches focus on different aspects of the modeling process. In this regard, MDE is appealing for addressing safety and security concerns where models play an integral role in describing and analyzing them.

Systematic mapping studies are meant to provide an overview of a research area through classification and counting contributions concerning the categories of that classification. They involve searching the available literature to know what topics have been covered and where the corresponding papers have been published.^7^ According to Kitchenham et al,^8^ the research questions (RQs) in mapping studies are general as they aim to discover research trends (e.g., publication trends over time and topics covered in the literature). This is in contrast to systematic reviews, which intend to aggregate evidence and hence formulate a particular goal (e.g., whether research results are practical and deployable for the industry).^9^ The outcome of a mapping study is an inventory of publications on the selected topic mapped to a classification.^10^ To sum up the difference, one can say that a systematic mapping study is a quantitative process where we try to assess the overall size and landscape of a specific research field. At the same time, a literature review is a qualitative assessment of a tiny part of the said landscape, and we try to find out how the soil underneath is made up.

This paper presents a systematic mapping study on the MDE of safety and security software systems. The overall aim of this study is to collect the relevant state of the art in this field of research. Besides that, we answer some crucial questions like frequently used methods and tools in this field, their applicable development stages, and in which application domains they have been evaluated. We also identify where the community prefers to publish research results and reveal recent publication trends in this field. We carefully selected 143 publications out of 27,259 relevant search results through a rigorous and systematic process. These publications were proven helpful in answering six judiciously crafted RQs, providing gainful insights, and identifying the directions for future research. Furthermore, it gives an overview of which topics in this research area have been more worked on, possible gaps, and an overall trend in the published works.

The article is organized as follows: Section 2 details the systematic mapping process, including the RQs we investigate in this study. Section 3 presents the results of the mapping study. Section 4 presents our analysis of the current state of the art. A brief overview of future research directions in this field is presented in Section 5. Section 6 discusses the threats to the validity of this study and the adopted mitigation strategies. Section 7 discusses the related work concerning similarities in both fields, cross‐fertilization, and other literature reviews. The paper is concluded in Section 8.

## 2 MAPPING STUDY PROCESS

### 2.1 Time period

We scope the time of related studies published from 1992 to 2020. The earliest paper in our mapping study was published in 1992, hence the starting time. We searched at the end of 2020, thus the ending time. The search was conducted again in Q1 of 2021 to ensure the completion of results for 2020.

### 2.2 Digital libraries

Five digital libraries were used in this mapping study: ACM, * IEEE, † Scopus, ‡ Springer, § and Web of Science. ¶ According to Chen at al.,^11^ these digital libraries are among the most popular sources in computer science and engineering that ensure a high coverage of potentially relevant studies. We did not include Google Scholar # in our mapping study as the search results of Google Scholar tend to be repetitive with respect to results from the included digital libraries, and its unique contribution to the search process is unclear.^11^

### 2.3 Tool

Conducting a systematic mapping study is a tedious and time‐consuming task. It usually involves the search, collection, filtration, and classification of many papers. Without a helping tool, this is a challenging endeavor. In this work, we used Zotero^12^ and spreadsheets. These tools helped us in importing, organizing, and analyzing search results. Python's Pandas ‖ and Plotly ** were used to provide the visualizations.

### 2.4 RQs

The goal of this mapping study (following the guidelines presented by Petersen et al.^7^, ^9^) is to discover what is the current state of the art in the field of MDE of safety and security software systems (and how it can be advanced in the future). This goal leads to the following precise RQs:

- RQ1: At which development stage the research was conducted?
  Rationale: MDE is a multistage development process. Therefore, we want to know at which development stage this research was conducted. Furthermore, this information can help us identify which development stages are susceptible to being focused more on engineering such systems.
- RQ2: Which methods and tools were employed during the research?
  Rationale: The use of methods and tools is inevitable during any research and development activity. This information can help us identify frequently used methods and tools for engineering such systems.
- RQ3: What is the classification of the research contribution?
  Rationale: We want to investigate the contribution type of articles through this question. According to Wieringa et al,^10^ contribution types refer to determining the type of intervention being studied. This could be a process, model, language, framework, and so on.
- RQ4: In which domain(s) research results were evaluated?
  Rationale: Safety and security systems may belong to various application domains, for example, railway, nuclear plants, and marine systems. We want to know the application domains in which the research results were evaluated by answering this question. This information can help us identify which application domains have gained more interest from developers of such systems.
- RQ5: Where was the research published?
  Rationale: By answering this question, we want to determine whether researchers prefer to publish in journals, magazines, conferences, or workshops. Usually, journals include more mature and concrete results, whereas conferences and workshops are targeted for timely discussion and early feedback. Thus, by answering this question, we can determine the maturity of the results in this field.
- RQ6: What is the research publication timeline and trend?
  Rationale: Timelines and publication trends tell us about the novelty and frequency of research. We can determine how the community is building around this area by answering this question. Is the topic a relatively new one, gaining popularity in recent years, or just phasing out? This information can help us determine the potential of this research topic.

### 2.5 Papers search and screening

**FIGURE 1:**
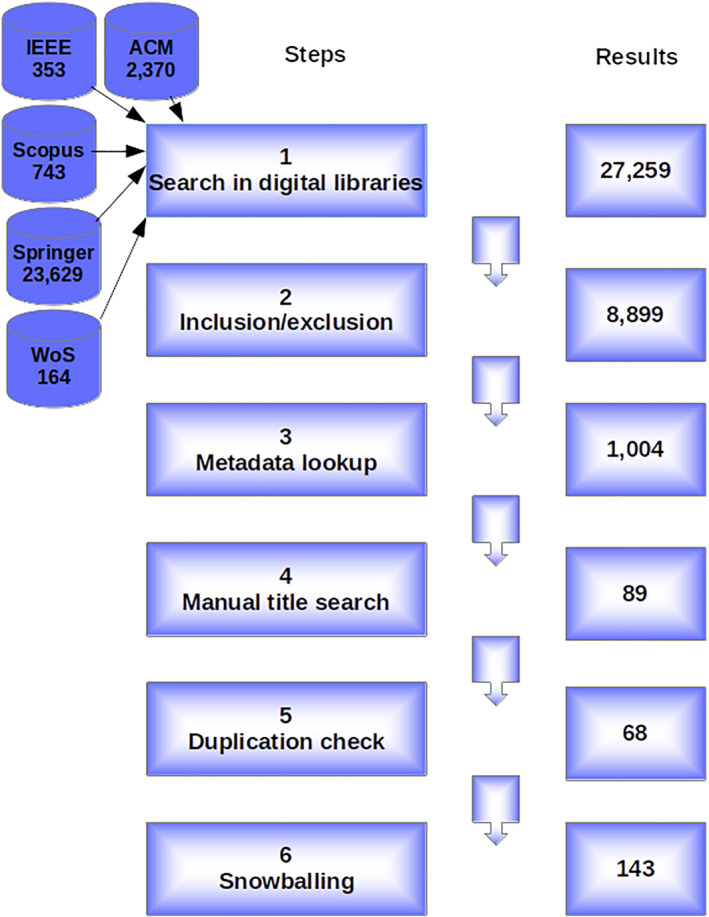
Steps for the search and selection process

The mapping study was conducted in six steps, as illustrated in Figure 1.

#### 2.5.1 Step 1—Search in digital libraries

The following query performed in the digital libraries produced 27,259 search results.

*(“model‐driven” OR “model‐based”) AND (“engineering” OR “development”) AND (“safety” OR “safe”) AND (“security” OR “secure”)*

Digital library‐wise acquired results are shown in Figure 1. The Springer digital library produced the maximum number of results, followed by the ACM Digital Library. The search process was simple in ACM, Scopus, Springer, and Web of Science digital libraries. The basic search fields were enough to run the query, which required no further processing. However, we had to use the advance search option in the IEEE Digital Library; we wrote the query in the command search and obtained the results.

The search terms were identified according to the study topic. Like Kitchenham and Charters,^13^ we adopted the Population, Intervention, Comparison, Outcomes (PICO) criteria to formulate the search terms.

- Population: According to Kitchenham and Charters,^13^ the population may refer to a specific software engineering role, a category of software engineers, an application area, or an industry group. In our case, population is the terms about “safety/safe” and “security/secure.”
- Intervention: According to Kitchenham and Charters,^13^ intervention may refer to a software methodology, a tool, a technology, or a procedure. In the context of this study, the intervention includes the terms “model‐driven” or “model‐based.”
- Comparison: The comparison part is not applicable in this mapping study because this mapping study does not involve the comparison of model‐driven and other types of approaches.
- Outcomes: Outcomes include the terms relevant to “engineering” or “development” activities.

We used the Boolean operator OR to join alternate words and synonyms in each part (i.e., population, intervention, and outcomes) and the Boolean operator AND to join the terms from the three parts, respectively.

#### 2.5.2 Step 2—Inclusion and exclusion of results

To make the study selection results objective, we defined the selection criteria employed in the study selection process. This brought down the overall result count from 27,259 to 8899 papers. The criteria are as follows:

Inclusion criteria:

- peer‐reviewed studies published in conferences, workshops, journals, magazines, or books;
- studies classified as computer science publications; and
- studies published in English.

Exclusion criteria:

- studies published as courses, newsletters, reports, reference work entries, and so on;
- studies not accessible in full text; and
- Studies presenting non‐peer‐reviewed results or gray literature.

#### 2.5.3 Step 3—Meta‐data lookup

We carefully checked the available meta‐data, for example, keywords and abstracts, of results in this step. First, we filtered out all those results that did not focus on “safety” and “security,” that is, they did not use those terms in their meta‐data. This brought down our results tally to 1004.

#### 2.5.4 Step 4—Manual title search

Despite the meta‐data lookup, some publications could still be included in the results, only remotely dealing with safety and security concerns. To ensure that the final result only includes high‐quality, relevant papers, we manually checked the title of each paper. We confirmed that each paper title has something to do with safety and security. As a result of this step, our result count became 89.

#### 2.5.5 Step 5—Check for duplicate results

Once we individually checked the results produced by each digital library, we merged them into a single repository. Because many digital libraries index the same venues, the search results may be redundant. To have a list of unique results, we check the list of merged results for duplicates in this step. Consequently, all duplicates were removed from the results list, and the publication count became 68.

#### 2.5.6 Step 6—Snowballing

In this last step, we performed snowballing readings. Snowballing refers to using the reference list of a paper or the citations to the paper to identify additional papers.^14^ For each paper identified for possible inclusion, we applied the same criteria employed to select papers in the first place. Then, we identified 75 further relevant studies in this step. After this, the final set of results reached the tally of 143 publications.

Because we performed both backward and forward snowballing, we discovered a relatively large number of new studies. However, a large number of found studies during snowballing is not so wrong because, according to Wohlin,^14^ the possibility of noise in snowballing is less than using a digital library approach, and, by deduction, snowballing is a better approach than a digital library search for extending literature studies.

### 2.6 Studies classification scheme

**FIGURE 2:**
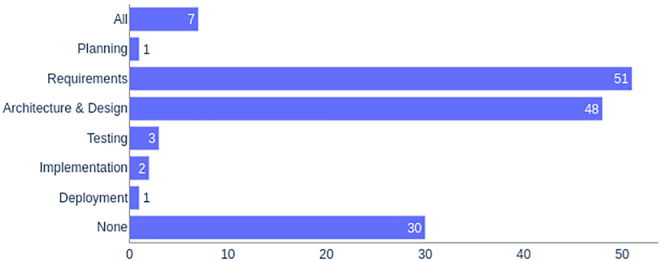
RQ1: Studies classification based on development stages

**FIGURE 3:**
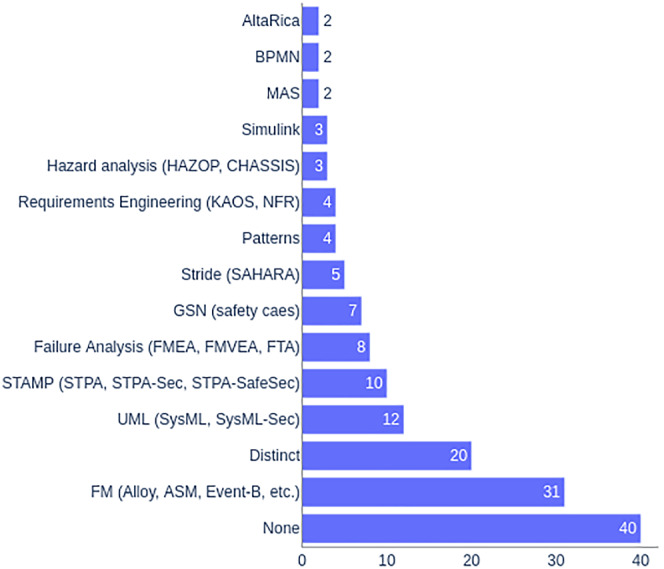
RQ2: Studies classification based on applied methods and tools

**FIGURE 4:**
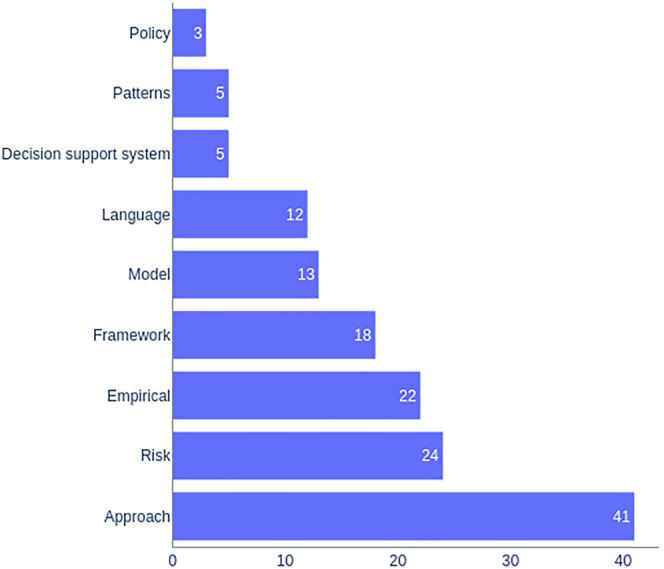
RQ3: Studies classification based on contributions

The classification scheme used for this study follows a systematic process suggested by Petersen et al.^7^, ^9^ We are using keywords as bases for studies classification. Initially, we read abstracts to find representative keywords and concepts. The set of extracted keywords from different studies are then unified to overview the nature and contribution of the research (e.g., as shown in Figure 3, studies might not use “formal methods” as a keyword, but the name of the method, e.g., “Alloy”). This would create a category for each formal method, and therefore, to avoid this situation, different formal methods were merged into a single category). This results in a set of categories representing the underlying population. Sometimes meaningful keywords could not be extracted from the abstract alone. In such cases, either introduction and conclusion sections were studied, or complete papers were skimmed through. Upon selecting the final set of keywords, they are clustered and consequently used to form the categories. Where applicable, classifications were also based on the Software Engineering Body of Knowledge (SWEBOK) † structure (e.g., as shown in Figure 2, which are the main life‐cycle activities of software engineering) or inspired from previous categorizations (e.g., as shown in Figure 4, which is based on the work of Wieringa et al.^10^).

To reduce any bias, we followed an iterative strategy. Three experienced researchers (listed as the first three authors of this article) participated in the study. Initially, the first author classified all primary studies as mentioned in Step 3 and Step 4 of Figure 1. The second author then reviewed the classifications and corrected them, where necessary, based on the meta‐data lookup (Step 3). In case of a disagreement, the third author independently reviewed the classification and judged. The opinion of the majority prevailed. However, the disagreement only occurred rarely. Although all participants of this study are senior and experienced researchers (while the first author has an industrial background, the second and third authors are university professors), this step involves human judgment. This again leads to the threat of bias, which cannot be eliminated entirely. This point is further elaborated in Section 6.

### 2.7 Data extraction and synthesis

To answer the RQs, we extracted specific data from selected publications. Table 1 describes data items that have been extracted in this mapping study.

**TABLE 1:** Extracted research items

Item name	Description	Relevant RQ
Development stage	At which development stage was the research applicable?	RQ1
Method/tool	What is the deployed method or tool in the research?	RQ2
Contribution classification	What is the classification of contribution of the research?	RQ3
Application domain	Which application domain the research was applied to?	RQ4
Publication type	What is the publication type of the research?	RQ5a
Publication venue	In which venue was the research published?	RQ5b
Publication year	In which year was the research published?	RQ6

Data synthesis targets to synthesize the extracted data to answer the RQs. The results of this task are discussed (also visually) in the following section.

## 3 MAPPING STUDY RESULTS

The results of the mapping study are shown in Table A1 (for convenience, located in Appendix A1) in chronological order. The whole table can also be accessed online. ‡‡ Please note that the names of publication venues are not listed here for brevity. Apart from the method/tool column, Table A1 contains the main categories of the entries, and subcategories are mentioned only in the discussion on the relevant RQ. We now synthesize the extracted data to answer previously listed RQs.

### 3.1 Development stages (RQ1)

Figure 2 shows the results of RQ1. Each study is counted only once in its respective category. “All” denotes studies covering the whole spectrum of MDE, that is, all development stages. “None” denotes studies that did not focus on any development stage.

Only a few studies (7 out of 143) covered the whole MDE spectrum, i.e., all development stages. Hassan et al^15^ were discussing the idea of how the Formal Analysis and Design for Engineering Security (FADES) approach can be used to support the model‐based software engineering (MBSE) paradigm. Bloomfield et al^16^ use the structured safety cases approach to discuss the impact that security might have on an existing safety case. Apvrille et al^17^ presented a similar idea based on the SysML‐Sec environment covering all the development stages. Pedroza^18^ presents a synthetic discussion on the safety and security topics along with some perspectives across all MDE stages. However, these studies did not use any application domain to evaluate the proposed approaches. The approach presented in Benyó et al,^19^ on the other hand, discussed the design and development of a smart card application management infrastructure by specifying business and technological processes and associated security requirements. Sabaliauskaite et al^20^ proposed the AVES framework for systematic model‐based analysis of safety and security properties of the automotive domain. Tang et al^21^ presented a domain‐specific modeling language to model smart home IoT applications and generated code out of the model.

Although the planning phase is crucial for the successful development of a system, only one study has focused on this stage. In this study, Park et al^22^ discuss how multiagent systems (MAS) and swarm intelligence can be exploited to boost counterterrorism and public safety activities using a rescue system example.

As anticipated, most of the studies (51 out of 143) were focusing on the level of requirements. Fifteen studies^23^, ^24^, ^25^, ^26^, ^27^, ^28^, ^29^, ^30^, ^31^, ^32^, ^33^, ^34^, ^35^, ^36^, ^37^ out of those 51 were focusing exclusively on requirements modeling of such systems. Thirty‐one studies^38^, ^39^, ^40^, ^41^, ^42^, ^43^, ^44^, ^45^, ^46^, ^47^, ^48^, ^49^, ^50^, ^51^, ^52^, ^53^, ^54^, ^55^, ^56^, ^57^, ^58^, ^59^, ^60^, ^61^, ^62^, ^63^, ^64^, ^65^, ^66^, ^67^, ^68^ were focusing on both requirements modeling and analysis. A few studies were either focusing solely on requirements analysis^69^, ^70^, ^71^, ^72^ or requirements traceability.^73^

The architecture and design stages are of paramount importance in the development of any system; safety and security systems are no exceptions. Forty‐eight out of 143 studies were focusing on this stage. Ten out of those studies^74^, ^75^, ^76^, ^77^, ^78^, ^79^, ^80^, ^81^, ^82^, ^83^ were discussing architecture modeling. Twenty‐four studies^84^, ^85^, ^86^, ^87^, ^88^, ^89^, ^90^, ^91^, ^92^, ^93^, ^94^, ^95^, ^96^, ^97^, ^98^, ^99^, ^100^, ^101^, ^102^, ^103^, ^104^, ^105^, ^106^, ^107^ were discussing architecture analysis. Fourteen studies^77^, ^108^, ^109^, ^110^, ^111^, ^112^, ^113^, ^114^, ^115^, ^116^, ^117^, ^118^, ^119^, ^120^ were discussing how to make architectural design of systems both safe and secure through modeling and analysis.

Testing also plays a pivotal role in the systems development life cycle. We found three studies focusing on testing in our mapping study. While Sojka et al^121^ were explicitly focusing on the testing of safety and security requirements within the automotive domain, Shahir et al^122^, ^123^ were focusing on test case generation for safety and security of marine systems.

Development stages, such as implementation,^124^, ^125^ and deployment and reconfiguration,^126^ were also mentioned in the literature; however, they were not the center of attention of researchers in this field.

Many studies (30 out of 143) did not focus on any development stage. Instead, they were either comparing safety and security concepts, for example, Burns et al,^127^ discussing how one can help achieve the other, for example, Brewer,^128^ making similarities and dissimilarities explicit between the two, for example, Blanquart et al,^129^ analyzing how the two concepts can cross‐fertilize each other, for example, Pietre et al^130^ and so on.

### 3.2 Methods and tools (RQ2)

Figure 3 graphically depicts the frequency of the applied methods and tools. Some approaches consisted of more than one method/tool. Papers that did not use any method are counted under “None.” Many papers used distinct methods, that is, appearing only in one study. These methods are shown in Figure 3 as “distinct.”

Our research shows that many methods and tools are used in this field, but none stands out. Although there is an observable tendency among the community to use formal methods for such kinds of engineering activities (31 studies are conducted using formal methods), no formal method can be classified as the method of choice. Among more frequently used formal methods, the use of Event‐B^33^, ^53^, ^56^, ^59^, ^62^, ^68^ and Abstract State Machines^76^, ^77^, ^108^, ^122^, ^123^ has been mentioned in six and five studies, respectively. The use of Alloy^48^, ^79^, ^112^ has been mentioned in three studies. However, all Abstract State Machines and Event‐B method applications stemmed from individual groups and targeted marine and control systems, respectively.

Unified Modeling Language (UML) and its variants, that is, Systems Modeling Language (SysML) and SysML‐Sec, are also relatively popular in this domain and found in 12 studies. The use of UML is mentioned in previous studies.^44^, ^66^, ^67^, ^82^ The use of SysML has been mentioned in previous studies.^30^, ^39^, ^64^ The use of SysML‐Sec (an extended version of the SysML language to design safe and secure embedded systems) has been found in previous studies.^17^, ^31^, ^70^, ^71^, ^72^

The third most widely used set of techniques was STAMP and its variants, that is, STPA, STPA‐Sec, and STPA‐SafeSec (10 studies). Systems‐Theoretic Accident Model and Processes (STAMP)^131^ is an accident causality model based on systems theory and systems thinking. Systems‐Theoretic Process Analysis (STPA)^132^ is a powerful hazard analysis technique based on STAMP. STPA‐Sec^111^ is a system‐theoretic process analysis method explicitly focusing on security issues. STPA‐SafeSec^120^ is an analysis methodology for both safety and security. The use of STAMP is mentioned in Troubitsyna et al.^33^ The use of STPA is mentioned in previous studies.^52^, ^58^, ^60^ The use of STPA‐Sec is mentioned in previous studies.^110^, ^111^, ^116^, ^117^, ^133^ The use of STPA‐SafeSec is mentioned in Friedberg et al.^120^

Failure analysis is the process of collecting and analyzing data to determine the cause of a possible failure in a system. Failure analysis methods (e.g., FMEA, FMVEA, and FTA) are commonly used to engineer safe and secure systems. Their use has been found in eight studies. The use of Failure Mode and Effects Analysis (FMEA) is mentioned in Hecht et al^64^ and Winther et al.^84^ The use of Failure Mode, Vulnerabilities, and Effects Analysis (FMVEA)—a variant of FMEA—has been found in previous studies.^89^, ^94^, ^99^, ^101^ The use of Fault Tree Analysis (FTA) has been found in Steiner and Liggesmeyer^86^ and Kornecki and Liu.^87^ An approach very similar to failure analysis is hazard analysis (e.g., HAZOP and CHASSIS). The use of hazard analysis approaches has been found in three studies; however, two were applied in combination with traditional failure analysis methods. The use of Hazard and Operability Study (HAZOP) in combination with FMEA is found in Winther et al^84^ and in combination with Combined Harm Assessment of Safety and Security for Information Systems (CHASSIS) has been found in Katta et al.^73^ The use of CHASSIS in combination with FMVEA has been found in Schmittner et al.^94^ Similarly, Security‐Aware Hazard Analysis and Risk Assessment (SAHARA) and STRIDE (an acronym for six security threat categories: spoofing, tampering, repudiation, information disclosure, denial of service, and elevation of privileges.) are hazard analysis and threat modeling approaches. The use of STRIDE is mentioned alone in Preschern et al^85^, ^100^ and in combination with SAHARA in Macher et al.^92^, ^93^ The use of SAHARA with FMVEA is mentioned in Dobaj et al.^99^

Another approach relatively popular in this domain is based on Goal Structuring Notation (GSN) and safety cases. The use of these notations has been found in seven studies.^34^, ^40^, ^41^, ^85^, ^100^, ^105^, ^117^

Goal‐oriented requirements engineering approaches, such as KAOS or NFR, also play an essential role in this domain. Their use has been found in four studies. The use of Knowledge Acquisition in Automated Specification (KAOS)—a goal‐oriented requirements engineering approach—has been found in Ponsard et al.^36^, ^51^ The use of the Non‐Functional Requirements (NFR) approach—a goal‐oriented technique that can be applied to determine the extent to which specific objectives are achieved by design—has been found in Kornecki et al^46^ and Subramanian and Zalewski.^95^

Some researchers have also proposed different patterns in this domain, that is, architectural safety patterns including security considerations,^85^, ^100^ safe & sec case patterns,^50^ and systems engineering patterns for interlinking safety and security.^119^

The use of Simulink—a graphical programming environment for modeling, simulating, and analyzing multidomain dynamical systems—has been found in three studies.^57^, ^103^, ^126^ Following are the methods whose use has been found in two studies apiece. The use of AltaRica—a high‐level language designed for the modeling of systems—has been mentioned in Bieber and Brunel^47^ and Brunel et al.^112^ The use of Business Process Model and Notation (BPMN)—a graphical representation for specifying business processes—has been found in Monakova et al.^42^, ^43^ Finally, the use of MAS has been found in Park et al^22^ and Poslad.^26^

Many found studies (40) did not employ any method or tool in the conducted research. Instead, they were either characterizing the differences between safety and security, for example, Burns et al.^127^; comparing the two approaches, for example, Raspotnig et al.^134^; stressing the need for their integration, for example, Eames et al.^24^; or demonstrating how they could complement each other, for example, Brewer et al.^128^

### 3.3 Contribution classification (RQ3)

As shown in Figure 4, most researchers of this domain are proposing an approach or a methodology based on an already existing method or tool, that is, 41 out of 143 publications.^26^, ^28^, ^33^, ^34^, ^35^, ^36^, ^40^, ^41^, ^46^, ^47^, ^48^, ^51^, ^52^, ^53^, ^56^, ^58^, ^59^, ^61^, ^62^, ^63^, ^64^, ^65^, ^66^, ^68^, ^71^, ^72^, ^74^, ^78^, ^82^, ^86^, ^87^, ^93^, ^95^, ^97^, ^98^, ^102^, ^114^, ^121^, ^126^, ^135^, ^136^ Their aim was to adapt an existing technology in such a way that it becomes exploitable for MDE of safety and security systems.

Many researchers were interested in deploying the MDE paradigm for risk‐related activities of safe and secure systems. Twenty‐four out of 143 studies focused on this aspect. While majority of researchers explicitly focused on risk analysis,^79^, ^84^, ^92^, ^94^, ^110^, ^111^, ^112^, ^113^, ^116^, ^117^, ^120^, ^133^, ^137^ some also focused on risk assessment,^16^, ^73^, ^91^, ^99^, ^101^ risk communication,^138^ risk management,^90^, ^118^ and risk modeling.^57^

Twenty‐two out of 143 studies had empirical contributions. Taxonomies and ontologies, which provide mappings of how various concerns overlap each other, were found three times.^32^, ^135^, ^136^ Surveys analyzing the challenges and possibilities for a combination of safety and security were found six times.^139^, ^140^, ^141^, ^142^, ^143^, ^144^ The concept papers that propose plans for a future application of a combined safety and security approach were found five times.^145^, ^146^, ^147^, ^148^, ^149^ The papers comparing safety and security regarding their similarities and differences were found five times.^127^, ^128^, ^129^, ^130^, ^134^ Two papers evangelize for a unified approach to safety and security.^150^, ^151^ One contribution compiled a small bibliography.^152^

Eighteen out of 143 studies presented a framework that could be useful in various phases of MDE of safety and security systems. A framework, in this context, means a platform providing a foundation for developing safety and security systems. These frameworks were based on either formal approaches,^27^, ^45^, ^49^, ^109^ SysML,^30^ or other various methods and tools.^19^, ^20^, ^22^, ^67^, ^69^, ^75^, ^81^, ^103^, ^104^, ^107^, ^151^, ^153^, ^154^

Thirteen out of 143 studies presented a model. The model could either be related to the collaborative modeling of safety and security properties,^147^ a life‐cycle model,^18^, ^25^, ^124^, ^155^ a process model,^24^, ^37^, ^44^, ^60^ a reference model,^55^ or a business process model^42^, ^43^, ^83^ for the development of safety and security systems.

Twelve out of 143 studies presented a language for the MDE of safety and security systems. The proposed languages were either formal languages,^15^, ^21^, ^38^, ^96^, ^115^ graphical modeling languages,^17^, ^31^, ^39^, ^70^ programming languages,^125^ architecture description language,^106^ or a language for presenting mishaps.^156^

Some researchers also presented some decision support systems,^76^, ^77^, ^108^, ^122^, ^123^ patterns,^50^, ^85^, ^100^, ^105^, ^119^ and policies^29^, ^54^, ^80^ for the MDE of safe and secure software systems.

### 3.4 Evaluation domains (RQ4)

**FIGURE 5:**
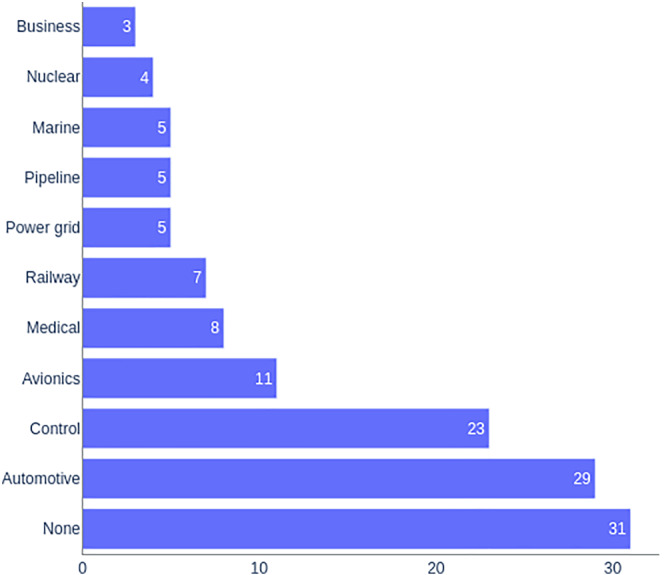
RQ4: Studies classification based on evaluation domains

Figure 5 graphically depicts the frequency of evaluation domains. Please note that some publications used more than one domain for evaluation purposes. Therefore, the papers belonging to multiple domains are counted multiple times.

Researchers dealing with safety and security were mostly interested in evaluating their proposed methodologies and tools in the automotive domain. Twenty‐nine^20^, ^31^, ^35^, ^51^, ^57^, ^58^, ^59^, ^61^, ^63^, ^65^, ^70^, ^82^, ^86^, ^89^, ^92^, ^93^, ^94^, ^103^, ^105^, ^116^, ^117^, ^119^, ^121^, ^141^, ^142^, ^147^, ^148^, ^151^, ^157^ studies were applied to automotive systems. After automotive systems, researchers of this field were mostly interested in applying their methods and tools in control systems. Twenty‐three studies were applied to control systems. Control systems included general‐purpose control systems,^39^, ^56^, ^62^, ^110^, ^124^, ^143^ air traffic control systems,^24^, ^44^, ^73^, ^102^ access control systems,^29^, ^54^ industrial control systems,^30^, ^33^, ^53^, ^80^, ^113^, ^144^ network control systems,^56^ electrical substation automation systems,^85^ and building automation systems.^25^, ^27^

After control systems, avionic/aviation systems were most attractive for researchers of this topic. This domain was used as a test bed in 11 studies.^47^, ^52^, ^67^, ^79^, ^87^, ^106^, ^109^, ^112^, ^133^, ^135^, ^136^

The use of medical systems has been mentioned in eight studies.^75^, ^81^, ^90^, ^98^, ^107^, ^109^, ^137^, ^153^ At the same time, the railway domain has been featured in seven studies.^32^, ^36^, ^45^, ^55^, ^72^, ^84^, ^140^

The use of the marine, pipeline, and power grid systems as an evaluation domain has been mentioned in five studies. The use of marine systems is mentioned in previous studies.^76^, ^77^, ^108^, ^122^, ^123^ However, an interesting point to note is that all these publications stemmed from a single group applying a particular method: Abstract State Machines. Pipeline systems, on the other hand, were mainly dealing with the oil industry.^46^, ^49^, ^88^, ^95^, ^96^ The use of power grid systems has been mentioned in previous studies.^69^, ^83^, ^104^, ^120^, ^126^

Nuclear systems were mentioned in four studies.^37^, ^66^, ^69^, ^91^

Business systems have been used as an evaluation domain in three publications. The use of business systems, mainly enterprise resource planning systems, has been mentioned in previous studies.^38^, ^42^, ^43^

Satellite and defense systems have been used as an evaluation domain in two publications each. The use of satellite systems is mentioned in Johnson and Yepez.^40^, ^41^ The use of defense systems is mentioned in Cockram and Lautieri^74^ and Cimatti et al.^115^

The use of fire detection,^48^ rescue,^22^ road transportation,^114^ smart card,^19^ smart cities,^146^ virtual organization,^26^ water supply,^64^ smart home,^21^ and voting^60^ systems has been mentioned only once in the found literature.

As aforementioned in Section 3.2, many found studies did not employ any particular method or tool in their research. Instead, these studies were either characterizing the differences between safety and security or stressing the need for their integration. Naturally, such comparative or road‐map studies were not always subject to evaluation. Consequently, many studies (31) we found were not evaluated on any particular domain.

### 3.5 Publication types and venues (RQ5)

#### 3.5.1 Publication types (RQ5a)

**FIGURE 6:**
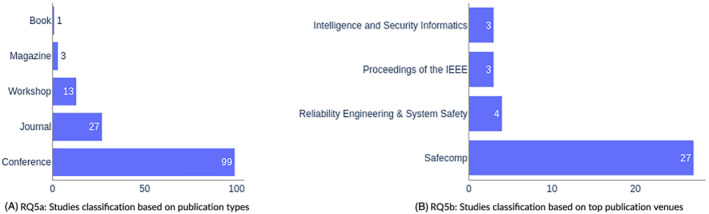
RQ5: Studies classification based on publication types and venues

Only peer‐reviewed publications (including books, journals, magazines, conferences, and workshops) were considered in this study. Please note that we did not consider books containing contributions by multiple authors. Such contributions are treated as independent studies. Figure 6A provides an overview of the distribution of studies between these venues. An overwhelming majority of studies (99/143) were published in conferences, followed by journals and workshops.

#### 3.5.2 Publication venues (RQ5b)

Looking at mapping study results, it was clear which venues were mostly targeted by researchers of this domain. The top 4 venues for this domain are shown in Figure 6B. The most favorite venue of researchers of this topic is undoubtedly the Conference on Computer Safety, Reliability, and Security (SAFECOMP). Twenty‐seven studies^24^, ^33^, ^47^, ^48^, ^50^, ^51^, ^52^, ^56^, ^68^, ^81^, ^84^, ^88^, ^89^, ^90^, ^92^, ^93^, ^99^, ^101^, ^103^, ^115^, ^116^, ^117^, ^119^, ^138^, ^141^, ^151^, ^157^ are published on this venue. Conference on Intelligence and Security Informatics (ISI),^77^, ^108^, ^122^ Proceedings of the IEEE,^109^, ^126^, ^149^ and Journal on Reliability Engineering & Systems Safety^130^, ^144^, ^154^ contained three publications each. Conference on Critical Information Infrastructures Security (CRITIS),^80^, ^139^ Journal on Security Informatics,^22^, ^123^ Conference on Emerging Technologies and Factory Automation (ETFA),^25^, ^143^ European Dependable Computing Conference (EDCC),^57^, ^59^ Symposium on Model‐Based Safety and Assessment (IMBSA),^62^, ^142^ Workshop on Interplay of Security, Safety and System/Software Architecture (ISSA),^18^, ^71^ and Workshop on Software Engineering for Resilient Systems (SERENE)^16^, ^53^ hosted two papers apiece. All other venues had only one publication each.

### 3.6 Publication timeline and trend (RQ6)

**FIGURE 7:**
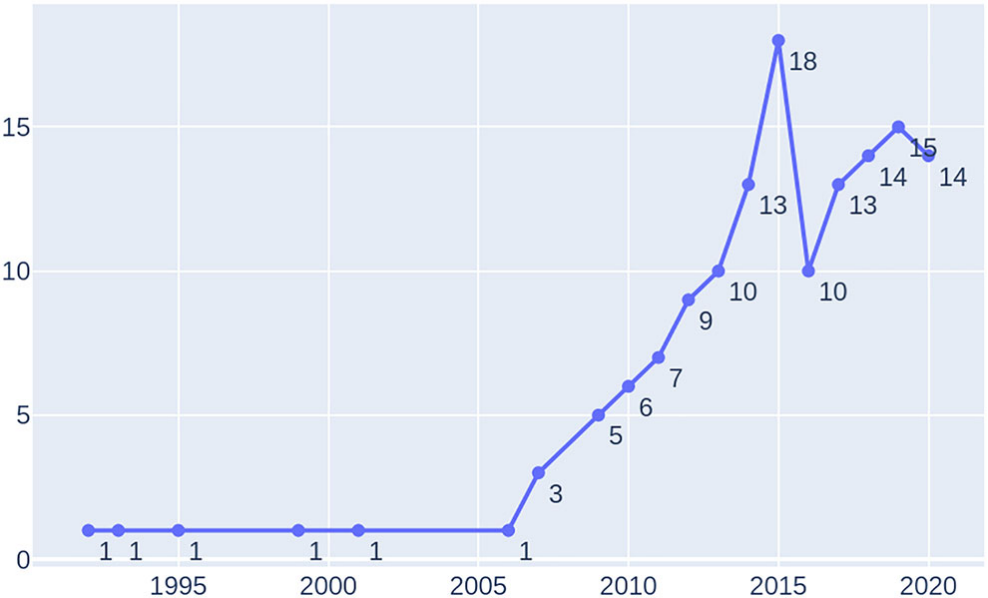
RQ6: Studies classification based on publication timeline and trend

Figure 7 shows the timeline and trend of publications in this area. As per our findings, the first study^127^ explicitly focusing on safety and security together was published in 1992. While the interest in this area was linear until 2006, a significant increase can be observed from 2007 onwards, reaching its top in 2015. Since then, like a typical hype cycle, the community is *perhaps* slowly climbing the “slope of enlightenment” towards the “plateau of productivity.” Nonetheless, the increase in the number of publications indicates that the area is considered highly relevant by the software engineering research community.

## 4 DISCUSSION AND INSIGHTS

*Regarding RQ1*, we have found that most researchers are working at the level of requirements or architecture. Modeling and analysis activities are the primary focus at both these levels. Only a few researchers consider the whole MDE spectrum (i.e., all development life‐cycle activities). While modeling and analysis of requirements and designs are essential activities, it is also imperative to ensure that these models are eventually translated into implementations as seamlessly as possible. Detailed works showing such transformations are currently missing from the state of the art and worth exploring in the future. Another critical point we observed is that testing is not a primary focus of researchers in this field. Although the code generated through a rigorous development process is, in principle, already verified and validated, this is not enough in the case of critical systems. For such systems, the generated code also needs to be tested.^158^ In our opinion, researchers in this field should give priority to testing as it uncovers different sets of problems than those found in earlier stages of development, for example, if the code is later manually modified to introduce further implementation details, the designer can use tests to check that no faults are introduced inadvertently. Other development stages, though important, like planning, implementation, and deployment, are also currently underrepresented. There is much room for applying model‐driven approaches in these areas to engineer safe and secure software systems.

*Regarding RQ2*, we have found out that no single method or tool is prevalent in this domain. Although formal approaches are common (which makes perfect sense given the critical nature of safety and security systems), no formal method stands out. Formal methods, such as Abstract State Machines or Event‐B, have been used to design and develop many systems. However, the use of these methods often stems from individual groups. The STAMP method—initially proposed for the safety domain—also looks promising in this field. Several STAMP variants have recently been proposed to extend its capability toward security systems. However, it needs to be applied to more domains and projects before its suitability for safety and security systems can be truly evaluated. Additionally, the current use of this method is also confined to modeling and analysis of requirements and design artifacts. In the future, applying this method (by extension) to other stages of development could be an exciting topic of research. Finally, the use of graphical modeling languages, such as UML or SysML, is lacking in this field; even the available work mainly concentrates on modeling and analysis of requirements. Given the potential of these languages, this could be a niche for further research to demonstrate their effectiveness through their widespread applications to safety and security systems.

*Regarding RQ3*, we have found out that most researchers were interested in the risk analysis of such systems. Risk analysis is a crucial activity in the domain of safety. This becomes even more crucial when safety is integrated with security. Various methods were used for risk analysis, and mostly, researchers worked at the architecture and design level. While hazard analysis (e.g., HAZOP) and failure analysis (e.g., FMEA or FTA) are already established methods for risk analysis, new approaches, such as FMVEA, STPA‐SafeSec, or SAHARA, are also emerging recently. Working towards maturity and improving these approaches by further application to new domains and projects is also an exciting research topic. Another interesting observation we made was that most researchers are extending the capabilities of existing methods and tools to solve the challenges of this field (e.g., FMVEA is based on well‐established FMEA or STPA‐SafeSec is based on popular STPA) rather than presenting new frameworks and languages. Of course, new pertinent frameworks (e.g., SAFESCALE^75^) or languages (e.g., FADES^15^) are also surfacing but relatively low in number. A few contributions were made in laying out the basic theoretic foundations of the field, including aligning methods for safety and security.

**FIGURE 8:**
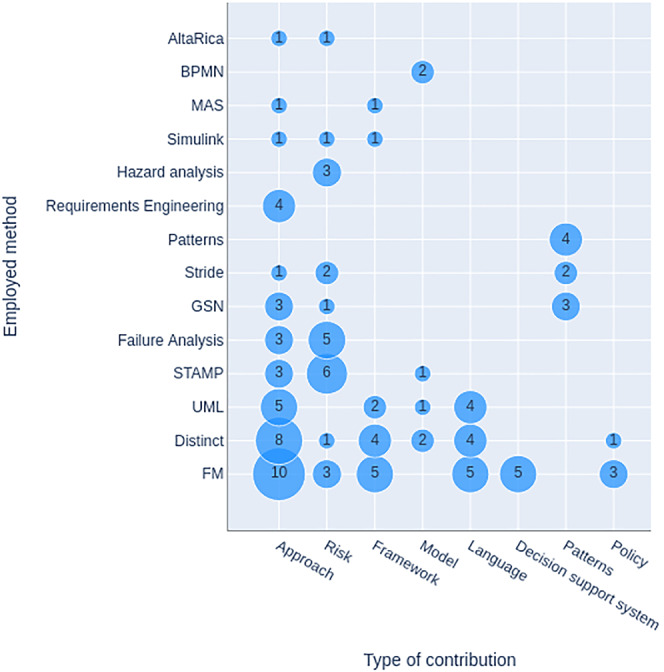
Overlapping between contribution types and their employed methods

In Figure 8, we show the focus points of the selected studies. On the vertical axis, there are categories of RQ2, that is, deployed methods and tools. On the horizontal axis, categories of RQ3 are used, that is, contribution types. Like in RQ2, entries can appear multiple times as one study can use numerous methods and tools, for example, as shown in the “patterns” column. Here, while the contribution type of studies is a pattern, they also used GSN and Stride methods. Nonetheless, what can be seen in Figure 8 is that formal methods got much attention in various areas. Most attention was given to finding an approach for realizing a system. This is not unsurprising as formal methods are already well established and well supported in the safety‐critical systems community. UML and its variants got second‐most attention, which is unsurprising, too, as this approach offers much flexibility. Other than that, most crossing points are barely or not populated, showing possible future research avenues.

*Regarding RQ4*, we have found out that most researchers used the automotive domain to evaluate their results. This is consistent with the emerging phenomenon of autonomous driving, where both safety and security play equally critical roles. However, the prominence of research in MDE for the automotive domain predates autonomous driving and has more to do with the adoption of this paradigm by the automotive industry.^159^ Control systems were also a favorite testbed for the evaluation of such systems. We believe two domains—medical and railway—are underrepresented in the current state of the art and should be further considered by the researchers in the future. Since medical systems have started becoming interoperable,^160^ cybersecurity has become an essential issue for these safety‐critical systems. Additionally, none of the found studies related to the medical domain focused on the requirements stage. This is an auspicious future research direction. Likewise, in the domain of railway, the advanced level of hybridness also necessitates the consideration of cybersecurity aspects.^161^ We will, therefore, most likely see catchup here when the technologies like ETCS Level 3^162^ are more and more adapted.

**FIGURE 9:**
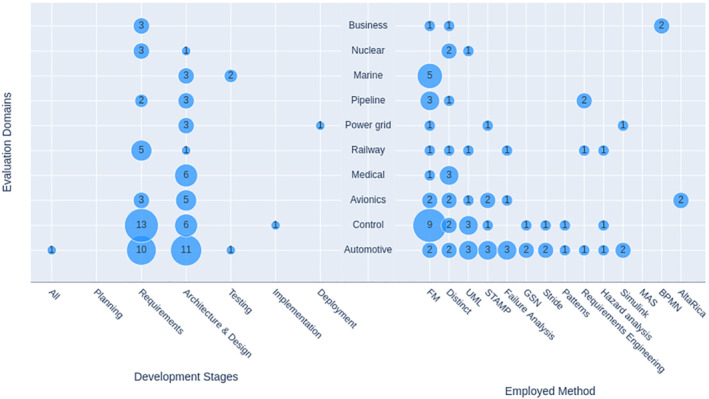
Overlapping between evaluation domains, development stages, and employed methods and tools

In Figure 9, we show how development stages and tool usage is distributed over the evaluation domains. Here, the planning stage has been included for completion even though it has no entry. The study related to the planning stage was evaluating rescue systems, which are not shown in Figure 9 due to the low frequency. The same goes for the label MAS. As already pointed out in RQ4, the automotive domain got much attention in the well‐populated requirements and architecture and design stages. However, the automotive row is evenly distributed when using methods and tools. Control systems got much attention in the formal methods category, which is not unsurprising. However, surprising is that the studies in the medical domain were conducted at the architecture stage (there are a total number of eight studies related to the medical domain. Six of those studies were conducted at the architecture stage, while two of them did not belong to any stage) and none of them with tools that are significant in number otherwise. The studies in the railway domain focus on requirements, but the employed methods and tools are spread out.

*Regarding RQ5*, we found that most researchers preferred to publish their results in conferences, especially in SAFECOMP. Articles appearing in journals were less in number and distributed among different venues. The numbers indicate that this research field is still (relatively) young and evolving. Also, more books must be published in this field to advance industrial maturity and adoption.

*Regarding RQ6*, we have found out that the community's interest is increasing in this research field. Moreover, more and more publications have explicitly focused on the MDE of safe and secure systems in the past few years. This temporal evolution is indeed suitable for its maturity and industrial uptake.

We have observed in our research that a significant number of publications did not mention any development stage, method, or evaluation domain in their results. This is mainly because these publications stressed the need for the joint modeling and development of safety and security by comparing the two concepts, discussing how one can help achieve the other, or analyzing how the two concepts can cross‐fertilize each other. So, naturally, such conceptual and road‐map studies were not subject to classification in respective RQs. Additionally, this large number of unclassified publications suggests the novelty of this field, that is, still much evangelizing is happening in this area.

## 5 FUTURE RESEARCH DIRECTIONS

Although we have given several hints in the preceding section, we would like to explicitly mention four areas where further research is required in this area. These hints are relatively broad as we think they offer promising ground‐laying future research direction. What we do not want to do is point out holes in the research we spotted in our analysis, like, for example, that there is no application of BPMN in the medical field, which one can see in Figure 9. This is because not every blank space is a promising research field. Reusing our example, BPMN is a business modeling technique, and its application in the medical domain that is highly safety‐critical might not even be feasible. Therefore, we concentrate on the bigger picture and point out the more prominent blank spots that might be interesting for a researcher to investigate.

### 5.1 Development of standards

The development of both safety and security systems is driven by standards today. Standards like IEC 61508 or ISO 26262 and ISO/IEC 27000 are already popular in safety and security domains, respectively. However, these standards do not offer any (concrete) technical advice on *combined* deployable processes and product qualities related to safety and security, even though they have a common origin in ISO 31000 regarding risk management. Furthermore, no *integrated* standard exists that addresses safety and security issues concurrently—especially the possible challenges emerging through their interplay; however, currently, two are under development to provide a bridge between both areas as described by Kanamaru.^155^ Further, the International Society for Automation (ISA) has formed a working group (Work Group 7 on cybersecurity and safety in industrial processes^163^) to investigate the potential coupling between safety and security. Nevertheless, as documented in the preliminary report,^163^ the group could not find a mathematical coupling between Safety Integrity Levels (SIL) and Security Levels (SL) due to the technical difference between the SIL and the SL calculation methods. Indeed, further efforts are required in this direction.

### 5.2 Cross‐fertilization among methods and tools

We have seen that formal approaches are among the frequently used methods and tools in this area. Indeed, state‐based formal methods^164^ seem to be quite suitable for the engineering of such kinds of systems: They cover all stages of the development life cycle, a variety of modeling and analysis tools are available at the disposal of developers, quality assurance is embedded within the development process, there is support for translation of requirements and design artifacts into correct pieces of software, and so on. The catch is that state‐based formal models may be opaque, that is, hard to read and write for many stakeholders.^165^, ^166^ Such developments can be augmented by using graphical modeling notations such as UML or SysML. This provides cross‐fertilization among various modeling tools and enables developers to harness the true potential of each tool at the suitable development stage. Some tools (e.g., UML‐B^167^) and approaches (e.g., KAOS‐Event‐B^168^) already exist and worth exploring in this regard. As far as risk analysis is concerned, which generally does not fall within the jurisdiction of formal methods, further research is required towards its harmonization with formal methods, such as shown by Khan et al.^169^ Another problem with state‐based formal methods is that while they offer practical tools for verification and validation, the support for automatic code generation is far from desirable.^170^ These methods can, in principle, generate code artifacts from models; however, the generated code needs much manual postprocessing. This may introduce some inconsistencies or errors in code, which may, in turn, compromise the integrity of the previously applied rigorous quality assurance process. This also makes systems susceptible to extensive testing, which is already a weak link in the development chain of such systems. Hence, future methods for MDE of safety and security systems need to offer better tools and methodologies, especially for code generation and testing, respectively. Looking from the security perspective, the STAMP method and its offshoots—being integrated approaches for safety and security—go beyond the risks mitigated by using formal methods, for example, human errors are also considered. The same is true for failure analysis. The approaches like FMEA aim to unify safety and security risk analysis. A unified analysis can ease the effort for the whole MDE process as, otherwise, multiple approaches for capturing safety and security may produce overlapping or contrary results.

### 5.3 Leveraging machine learning

We have observed a limited, relatively nonexistent use of machine learning in MDE of safety and security systems while conducting this study. However, because various machine learning techniques have been successfully deployed for system safety and security through machine vision and digital image processing, we believe the MDE community can also benefit from this. The power of machine learning combined with the agility of MDE can undoubtedly facilitate the development of safety and security software systems.

### 5.4 Further application domains

As shown previously, most researchers focused on automotive systems to evaluate their results, followed by control systems. We believe several other domains, such as the medical and railway sectors, deserve equal attention, currently underrepresented. Primarily, none of the studies related to the medical domain focused on the requirements stage. This is an auspicious future research direction. Security is lately also becoming an essential phenomenon in these traditional safety‐critical domains, and there is a huge potential for experimentation and advancing the state of the art. Another futuristic domain consists of smart systems such as smart grids and smart cities. For security as a stand‐alone topic, there are already very high‐level standards in use, for example, the NIS Directive of the European Union Agency for Cybersecurity. §§ This standard aims to harmonize the cybersecurity solutions of the members and enhance cross‐border collaboration in this field. The standard is used for critical infrastructures like banking, water supply, health, energy, and digital services. In addition, there is a scope for its extension to further domains such as railways and automotive.

## 6 THREATS TO VALIDITY

There is always a threat of validity for such kinds of empirical studies. We also face several threats in our systematic mapping process, which we discuss as follows. The categorization is taken from Zhou et al.^171^

### 6.1 Internal validity

As found by Petersen et al,^9^ quality assessment is not common in mapping studies. This is also consistent with suggestions of Kitchenham et al,^8^ which state that quality assessment is not essential for mapping studies as their overall aim is to give a broad overview of the topic area. However, despite these observations, we have adopted a rigorous process for inclusion/exclusion and classification of papers, which ensures that only high‐quality‐related papers are selected as primary studies. Another internal validity threat is regarding the source of the data. We used five digital libraries as a primary source for this research. All selected digital libraries are well known in the computer science discipline for including the most relevant results.^172^ Additionally, Wohlin et al^173^ state that having a more extensive set of papers is not necessarily better for mapping studies. The important thing is that found studies are a good representation of the population, which we ensured in this study by adopting a rigorous paper selection process.

### 6.2 Construct validity

The RQs themselves can be a threat: Are they the right kind of questions we should be asking? To minimize this threat, we judiciously crafted the questions in alignment with the overall aim of this work after having several internal discussions. The final set of RQs reflects our work's goals of providing an overview of this field's current state‐of‐the‐art and future research directions. Another threat to the integrity of the study is related to the terms used in search queries. To minimize this threat, we adopted the PICO criteria^13^ to formulate the search terms. The selected terms unequivocally represent the goals of our work. We discovered many publications during snowballing because we employed an extensive snowballing process, including backward and forward snowballing. However, many found studies in snowballing are not flawed as the possibility of noise in snowballing is less than using a digital library approach.^14^ An associated issue is the frequently used acronyms for model‐based/driven engineering. Although the query used did not explicitly include related acronyms, such as MDE, model‐driven development (MDD), or MBSE, this would not result in missing relevant articles because such information is usually available (or redundant) in meta‐data, for example, keywords or index terms, hence accessible.

### 6.3 Conclusion validity

As gray literature was ruled out from the beginning of the study, the results can be biased, especially regarding the implementation and application aspect of MDE for safety and security. We see, however, difficulties with including gray literature as the process of its selection is highly biased. First, as these types of publications are not listed in databases, a specific search must be conducted where vital results might not be found, leading to a false representation of reality. Second, companies tend to only publish success stories due to their market interest. This could also heavily bias the results of the study. We aim to provide a map of research progress, showing which areas are already investigated and which are not. Software success stories would populate certain areas while leaving those out where companies were unsuccessful and distort how the research areas are indeed populated.

## 7 RELATED WORK

Some authors have already presented initial findings on the available literature about safety and security systems. For example, Chockalingam et al^139^ present a survey on integrated safety and security risk assessments methods and their application domains. Abulamddi^174^ additionally caters to extracting requirements from the found risks. However, these works are limited to risk assessment and requirement extraction, just one aspect of our broader study. Piètre‐Cambacédès and Bouissou^130^ provide a survey on similarities and differences between safety and security approaches, including their interplay. The authors identify cross‐fertilization between the two areas and how the method from one area can be utilized in the other. A very narrow view of this cross‐fertilization is discussed within the industrial control applications in the work of Kriaa et al.^144^ Another work on cross‐fertilization, focusing on common standards and approaches to deeper entangle safety and security, is from Ponsard et al.^175^ In contrast to these works, our study focuses on the *MDE* of safety and security systems, what are the proposed methods and tools for each development stage, and what are various types of contributions in this regard.

A recently conducted systematic literature review on safety and security co‐analyses is presented by Lisova et al.^176^ In contrast to our study, (1) this work is only focusing on the requirements engineering stage, (2) this work focuses on a short span of publication timeline between 2012 and 2017, and (3) a smaller number of digital libraries are consulted in this study, hence a relatively limited set of papers available for analysis. Additionally, the nature of the RQs we are answering in our study is much broader and covers a broader spectrum of the area. Finally, as aforementioned, the nature of systematic literature reviews and systematic mapping studies are fundamentally different.

## 8 CONCLUSION

This article presents a systematic mapping study on MDE of safety and security software systems. Our mapping study provides an overview of the current state of the art in this field. Through a rigorous and systematic process, this study carefully selected 143 publications out of 27,259 relevant search results, which proved very helpful in answering the judiciously crafted RQs like the frequently used methods and tools, the important life‐cycle development stages, and the frequently used evaluation domains. Additionally, we identified the community's preference for publication venues and publication trends. Finally, based on the analysis of selected studies, we indicated several avenues for future research.

The current state of the art provides practical support for modeling and analysis of requirements and design of safety and security software systems. However, the state of the art needs to be advanced to offer better tools and methodologies, especially for code generation and testing. Better integration of graphical modeling languages with conventional formal notations and harmonizing rigorous methods and risk analysis approaches will also help. We also welcome more studies encapsulating the whole spectrum of MDE applied to safety and security systems, significantly leveraging machine learning approaches. Standards specific to the interplay between safety and security are also missing and need to be focused on soon.

In the future, we want to extend this study by asking qualitative questions like what the maturity level of the presented contribution is, how useful it is for the given task, which impetuses are required as input, and whether the contribution is applicable at the design time (static) or at the run time (dynamic). Regarding the MDE approach, it would be helpful to know the substantial aspects of developing safe and secure systems and the best way to apply MDE in the development. Analogously, machine learning, which we identified as a future research aspect, offers an opportunity for deeper investigation and research. Questions might be how machine learning influences the MDE aspect or helps develop safe and secure software systems. As assessing safety and security threads is a complex task, it might be worth considering them as multiple‐criteria problems. A modeler can tackle these problems with multiple‐criteria decision‐making (MCDM) techniques like, for example, MEW^177^ where multiple criteria might conflict with each other. A modeler can then decide trade‐offs with the help of fuzzy mathematics. However, it might be worth discussing how relevant this is for the safety‐security domain. The finished product needs to fulfill standards for both aspects to get clearance for operating the device. This might be a venture for further investigation.

## Data Availability

The data that support the findings of this study are openly available in Zenodo at https://doi.org/10.5281/zenodo.5785657.

## Footnotes

*
https://dl.acm.org.

†
https://ieeexplore.ieee.org/Xplore/home.jsp.

‡
https://www.scopus.com.

§
https://link.springer.com.

¶
https://apps.webofknowledge.com.

#
https://scholar.google.com.

‖
https://pandas.pydata.org/.

**
https://plotly.com/python/.

†
https://www.computer.org/education/bodies-of-knowledge/software-engineering.

‡‡
https://doi.org/10.5281/zenodo.5785657.

§§
https://www.enisa.europa.eu/topics/nis-directive.

## 1

**TABLE A1:** Classification map

#	Publication	Development stage	Method/tool	Contribution type	Evaluation domain	Publication type	Year
1	^127^	None	None	Assorted	None	Journal	1992
2	^128^	None	None	Assorted	None	Conference	1993
3	^23^	Requirements	None	Assorted	None	Conference	1995
4	^24^	Requirements	None	Model	None	Conference	1999
5	^84^	Design	HAZOP‐FMEA	Risk analysis	Railway system	Conference	2001
6	^156^	None	Mishaps	Language	None	Journal	2006
7	^154^	None	None	Framework	None	Journal	2007
8	^74^	Design	SafSec	Approach	Defense system	Conference	2007
9	^25^	Requirements	None	Model	Automation system	Conference	2007
10	^145^	None	None	Assorted	None	Conference	2009
11	^143^	None	None	Assorted	Control system	Conference	2009
12	^26^	Requirements	MAS	Approach	Virtual organization	Conference	2009
13	^27^	Requirements	Formal methods	Framework	Automation system	Workshop	2009
14	^75^	Design	SAFESCALE	Framework	Medical system	Conference	2009
15	^38^	Requirements	HRU	Language	Business system	Conference	2010
16	^76^	Design	ASMs	Decision support	Marine system	Conference	2010
17	^77^	Design	ASMs	Decision support	Marine system	Conference	2010
18	^15^	All	FADES	Language	None	Workshop	2010
19	^28^	Requirements	BDMP	Approach	None	Conference	2010
20	^69^	Requirements	SEMA	Framework	Power grid/Nuclear	Journal	2010
21	^29^	Requirements	HRU	Policy	Control system	Conference	2011
22	^108^	Design	ASMs	Decision support	Marine system	Conference	2011
23	^40^	Requirements	Safety cases	Approach	Satellite system	Conference	2011
24	^41^	Requirements	Safety cases	Approach	Satellite system	Conference	2011
25	^39^	Requirements	SysML	Language	Control system	Conference	2011
26	^122^	Testing	ASMs	Decision support	Marine system	Conference	2011
27	^78^	Design	Markov model	Approach	None	Conference	2011
28	^109^	Design	Formal methods	Framework	Medical/Avionics	Journal	2012
29	^129^	None	None	Assorted	None	Conference	2012
30	^45^	Requirements	LTL	Framework	Railways system	Journal	2012
31	^124^	Implementation	None	Model	Control system	Book	2012
32	^42^	Requirements	BPMN	Model	Business system	Conference	2012
33	^43^	Requirements	BPMN	Model	Business system	Conference	2012
34	^22^	Planning	MAS	Framework	Rescue system	Journal	2012
35	^44^	Requirements	UML	Model	Control system	Conference	2012
36	^123^	Testing	ASMs	Decision support	Marine system	Journal	2012
37	^16^	All	Safety cases	Risk analysis	None	Workshop	2013
38	^73^	Requirements	HAZOP/CHASSIS	Risk analysis	Control system	Conference	2013
39	^87^	Design	FTA	Approach	Aviation system	Journal	2013
40	^46^	Requirements	NFR	Approach	Pipelines system	Conference	2013
41	^30^	Requirements	SysML	Framework	Control system	Conference	2013
42	^130^	None	None	Assorted	None	Journal	2013
43	^85^	Design	STRIDE/Patterns	Pattern	Automation system	Conference	2013
44	^134^	None	None	Assorted	None	Journal	2013
45	^86^	Design	FTA	Approach	Automotive system	Conference	2013
46	^110^	Design	STPA‐Sec	Risk analysis	Control system	Conference	2013
47	^47^	Requirements	AltaRica	Approach	Avionics system	Conference	2014
48	^79^	Design	AltaRica/Alloy	Risk analysis	Avionic system	Workshop	2014
49	^112^	Design	Alloy	Risk analysis	Avionic system	Workshop	2014
50	^91^	Design	USSRAM	Risk analysis	Nuclear system	Conference	2014
51	^146^	None	None	Assorted	Smart cities	Journal	2014
52	^138^	None	None	Risk analysis	Automation system	Conference	2014
53	^88^	Design	BDMP	Risk analysis	Pipeline system	Conference	2014
54	^135^	None	None	Approach	Avionics	Conference	2014
55	^147^	None	None	Model	Automotive system	Journal	2014
56	^89^	Design	FMVEA	Risk analysis	Automotive system	Conference	2014
57	^121^	Testing	AUTOSAR	Approach	Automotive system	Conference	2014
58	^90^	Design	None	Risk analysis	Medical system	Conference	2014
59	^111^	Design	STPA‐Sec	Risk analysis	Medical system	Magazine	2014
60	^31^	Requirements	SysML‐Sec	Language	Automotive system	Conference	2015
61	^136^	None	COM	Approach	Aviation	Conference	2015
62	^48^	Requirements	Alloy	Approach	Fire detection system	Conference	2015
63	^114^	Design	EAST‐ADL	Approach	Road transport system	Conference	2015
64	^115^	Design	OCRA	Language	Defense system	Conference	2015
65	^32^	Requirements	None	Assorted	Railway system	Journal	2015
66	^113^	Design	S‐Cube	Risk analysis	Control system	Journal	2015
67	^144^	None	None	Assorted	Control system	Journal	2015
68	^49^	Design	Formal methods	Framework	Pipeline system	Conference	2015
69	^92^	Design	STRIDE/SAHARA	Risk analysis	Automotive system	Conference	2015
70	^93^	Design	STRIDE/SAHARA	Risk analysis	Automotive system	Conference	2015
71	^152^	None	None	Assorted	None	Journal	2015
72	^70^	Requirements	SysML‐Sec	Language	Automotive system	Conference	2015
73	^150^	None	None	Assorted	None	Conference	2015
74	^151^	None	None	Framework	Automotive system	Conference	2015
75	^94^	Design	FMVEA‐CHASSIS	Risk analysis	Automotive system	workshop	2015
76	^50^	Requirements	Patterns	Pattern	None	Conference	2015
77	^153^	None	None	Framework	Medical	Conference	2015
78	^17^	All	SysML‐Sec	Language	None	Conference	2016
79	^19^	All	None	Framework	Smart card system	Conference	2016
80	^51^	Requirements	KAOS	Approach	Automotive system	Conference	2016
81	^116^	Design	STPA‐Sec	Risk analysis	Automotive system	Conference	2016
82	^148^	None	None	Assorted	Automotive system	Conference	2016
83	^95^	Design	NFR	Approach	Pipeline system	Journal	2016
84	^34^	Requirements	STAMP/Safety cases	Approach	None	Conference	2016
85	^33^	Requirements	Event‐B	Approach	Control system	Conference	2016
86	^80^	Design	LTL/CFG	Policy	Control system	Conference	2016
87	^137^	Design	LTL/CFG	Policy	Control system	Conference	2016
88	^119^	Design	Patterns	Pattern	Automotive system	Conference	2017
89	^54^	Requirements	HRU	Policy	Control system	Conference	2017
90	^35^	Requirements	None	Approach	Automotive system	Conference	2017
91	^139^	None	None	Assorted	None	Conference	2017
92	^120^	Design	STPA‐SafeSec	Risk analysis	Power grid	Journal	2017
93	^118^	Design	PEARS	Risk analysis	None	Journal	2017
94	^155^	None	None	Model	None	Conference	2017
95	^96^	Design	AFT	Language	Pipeline system	Conference	2017
96	^117^	Design	STPA‐Sec	Risk analysis	Automotive system	Conference	2017
97	^140^	None	None	Assorted	Railway system	Conference	2017
98	^52^	Requirements	STPA	Approach	Avionics system	Conference	2017
99	^53^	Requirements	Event‐B	Approach	Control system	Workshop	2017
100	^149^	None	None	Assorted	None	Journal	2017
101	^125^	Implementation	C	Language	None	Conference	2018
102	^178^	None	None	Assorted	None	Conference	2018
103	^179^	None	None	Assorted	None	Magazine	2018
104	^141^	None	None	Assorted	Automotive system	Conference	2018
105	^55^	Requirements	SSIRM	Model	Railway system	Conference	2018
106	^36^	Requirements	KAOS	Approach	Railway system	Workshop	2018
107	^58^	Requirements	STPA	Approach	Automotive system	Journal	2018
108	^57^	Requirements	Simulink	Risk analysis	Automotive system	Conference	2018
109	^157^	None	None	Assorted	Automotive system	Conference	2018
110	^97^	Design	None	Approach	Control system	Journal	2018
111	^126^	Deployment	Simulink	Approach	Power grid	Journal	2018
112	^56^	Requirements	Event‐B	Approach	Control system	Conference	2018
113	^59^	Requirements	Event‐B	Approach	Automotive system	Conference	2018
114	^98^	Design	None	Approach	Medical system	Conference	2018
115	^71^	Requirements	SysML‐Sec	Approach	None	Workshop	2019
116	^60^	Requirements	STPA	Model	Voting system	Conference	2019
117	^99^	Design	SAHARA/FMVEA	Risk analysis	None	Conference	2019
118	^102^	Design	Formal methods	Approach	Control system	Conference	2019
119	^61^	Requirements	UPPAAL	Approach	Automotive system	Conference	2019
120	^81^	Design	SSAF	Framework	Medical system	Conference	2019
121	^142^	None	None	Assorted	Automotive system	Conference	2019
122	^37^	Requirements	HLIM	Model	Nuclear system	Conference	2019
123	^18^	All	None	Model	None	Workshop	2019
124	^133^	Design	STPA‐Sec	Risk analysis	Aviation system	Journal	2019
125	^100^	Design	STRIDE/Patterns	Pattern	None	Journal	2019
126	^20^	All	AVES	Framework	Automotive system	Conference	2019
127	^101^	Design	FTA/FMVEA	Risk analysis	None	Conference	2019
128	^62^	Requirements	Event‐B	Approach	Control system	Conference	2019
129	^82^	Design	UML	Approach	Automotive	Conference	2019
130	^63^	Requirements	Common criteria	Approach	Automotive system	Workshop	2020
131	^103^	Design	Simulink	Framework	Automotive system	Workshop	2020
132	^64^	Requirements	SysML/FMEA	Approach	Water supply system	Conference	2020
133	^65^	Requirements	STORM	Approach	Automotive system	Conference	2020
134	^104^	Design	UPPAAL	Framework	Power grid system	Conference	2020
135	^105^	Design	GSN	Pattern	Automotive system	Journal	2020
136	^66^	Requirements	UML	Approach	Nuclear system	Conference	2020
137	^67^	Requirements	UML	Framework	Avionic system	Conference	2020
138	^68^	Requirements	Event‐B	Approach	Control system	Workshop	2020
139	^106^	Design	AADL	Language	Aviation system	Magazine	2020
140	^107^	Design	PRF	Framework	Medical system	Conference	2020
141	^83^	Design	None	Model	Power grid system	Conference	2020
142	^21^	All	Lustre	Language	Smart home system	Conference	2020
143	^72^	Requirements	SysML‐Sec	Approach	Railway system	Conference	2020
